# Nonlinear dose–response relationship between dietary inflammatory index and risk of depression: a systematic review and meta-analysis

**DOI:** 10.3389/fnut.2025.1645789

**Published:** 2025-09-12

**Authors:** Lirong Yu, Lingzi Bian, Liping Ren, Wei Wei, Huijie Zhang, Maoyun Miao

**Affiliations:** ^1^School of Nursing, Shandong Second Medical University, Weifang, China; ^2^Qingdao Stomatological Hospital Affiliated to Qingdao University, Qingdao, China; ^3^Affiliated Hospital of Shandong Second Medical University, Weifang, China

**Keywords:** dietary inflammatory index, diet, depression, dose–response relationship, systematic review, meta-analysis

## Abstract

**Background:**

Depression is a prevalent mental health disorders that impose a significant global health burden. Emerging evidence suggests that diet plays a critical role in mental health, primarily through its impact on inflammation. The Dietary Inflammatory Index (DII) is a validated tool designed to assess the inflammatory potential of an individual’s diet.

**Objective:**

To systematically evaluate the association between DII and the risk of depression.

**Methods:**

A comprehensive search was conducted in PubMed, Cochrane Library, Embase, and Web of Science from inception to August 9, 2025. Two independent reviewers screened the studies, extracted data, and assessed methodological quality. A meta-analysis was performed to evaluate the association between DII and depression (the main outcome). The dose–response relationship between DII and depression was further analyzed using generalized least squares estimation and restricted cubic spline models in Stata 18.0.

**Results:**

A total of 43 studies were included. The meta-analysis revealed that higher DII scores were associated with an increased risk of depression (OR = 1.53; 95% CI: 1.42 to 1.66; *I*^2^ = 81.5%). Subgroup analyses stratified by study design, gender, age, region, dietary assessment methods, depression assessment tools, and body mass index (BMI) consistently showed a positive association between higher DII and depression risk. Dose–response analysis indicated a nonlinear relationship (*p* = 0.0019): no significant association was observed for DII scores below 0, whereas the risk increased progressively for scores above 0. Exploratory analyses of a smaller subset of studies suggested a similar trend for anxiety, but this finding should be interpreted with caution.

**Conclusion:**

Higher DII scores are associated with an increased risk of depression. These results highlight the potential benefits of reducing pro-inflammatory dietary components and encouraging anti-inflammatory eating patterns to support mental health, particularly in the prevention of depression.

**Systematic Review Registration:**

https://www.crd.york.ac.uk/PROSPERO/view/CRD42023433767, identifier (CRD42023433767).

## 1 Introduction

Depression is a prevalent mental health condition characterized primarily by a persistent low mood. It is estimated that approximately 350 million individuals worldwide suffer from depression, making it the leading cause of disability globally (1, 2). Anxiety, defined as a negative emotional response to perceived threats or stressors, has a lifetime prevalence of 7.3% in China (3, 4). Both depression and anxiety rank among the top 10 contributors to the global burden of disease (5), significantly reducing quality of life and, in some cases, leading to severe consequences such as self-harm and suicide. These disorders profoundly affect individuals’ work and daily functioning, placing a considerable economic burden on society (6, 7). Although current treatment options, including pharmacotherapy and cognitive behavioral therapy, are moderately effective, they are often associated with limited long-term efficacy, treatment instability, and high relapse rates (8). Therefore, there is an urgent need to explore novel preventive and therapeutic strategies.

In recent years, increasing attention has been given to the role of diet in mental health, particularly its influence on systemic inflammation. Chronic inflammation is believed to be a key mechanism underlying the development of various psychiatric conditions, including depression and anxiety (9, 10). Diet is a modifiable factor that can either amplify or alleviate inflammation. The concept of dietary inflammation has therefore gained prominence in mental health research. Meta-analyses of dietary patterns suggest that anti-inflammatory diets, such as the Mediterranean diet, or those with lower inflammatory scores, are associated with a reduced risk of depression (11).

The Dietary Inflammatory Index (DII), initially developed by James et al. in 2009 and later refined by Shivappa et al., is a composite scoring system designed to quantify the inflammatory potential of a person’s diet. It incorporates 36 anti-inflammatory and 9 pro-inflammatory food parameters (12, 13). Unlike studies that focus on individual nutrients or food groups, the DII evaluates the overall inflammatory potential of the diet, offering a more holistic assessment of how dietary patterns affect health (14).

A higher DII score indicates a more pro-inflammatory diet, while a lower score reflects an anti-inflammatory diet, similar in composition to the Mediterranean diet (12). As a validated tool for evaluating dietary inflammation, the DII has significant potential in guiding dietary recommendations, reducing systemic inflammation, and lowering the risk of chronic diseases (12, 13).

The association between DII and mental health outcomes has become an area of growing interest. Emerging evidence suggests that elevated DII scores are linked to an increased risk of both depression and anxiety (15). However, due to variability in study design, populations, and geographic settings, findings have been inconsistent, and the dose–response relationship between DII and mental health outcomes remains unclear.

This study aims to systematically synthesize the existing evidence through a dose–response meta-analysis to evaluate the association between DII and the risk of depression. By quantifying the impact of varying dietary inflammation levels on mental health, the research seeks to inform public health strategies and dietary interventions aimed at reducing the burden of mood disorders.

## 2 Methods

This study followed the PRISMA guidelines for systematic reviews and meta-analyses (16). The review protocol was registered in the PROSPERO database (Registration ID: CRD 42023433767). The completed PRISMA checklist is provided in (Supplementary File 3).

### 2.1 Search strategy

A systematic search was conducted using a combination of Medical Subject Headings (MeSH) and free-text terms across four databases: PubMed, Cochrane Library, Embase, and Web of Science. The search period extended from database inception to August 9, 2025. To ensure comprehensive coverage, the reference lists of all included articles were reviewed for additional relevant articles.

The English search terms included: “Dietary inflammatory index,” “DII,” “Inflammatory diet,” “Anti-inflammatory diet,” “Dietary score,” “Depression,” “Depressive symptom,” “Symptom depressive,” “Anxiety,” “Angst,” and “Nervousness.” The detailed search strategy is provided in (Supplementary File 2).

### 2.2 Selection criteria

Studies were eligible for inclusion if they met the following criteria: (1) Observational design, including cross-sectional, cohort, or case–control studies; (2) Assessment of the DII as a categorical variable; (3) Primary outcomes related to depression or anxiety symptoms; (4) Reported effect size estimates with 95% confidence intervals (CIs), such as odds ratios (ORs), relative risks (RRs), or hazard ratios (HRs).

Studies were excluded if they met any of the following conditions: (1) Involved pregnant or postpartum women participants; (2) Published in a non-English language; (3) Lacked full-text availability or were duplicate publications; (4) Did not report extractable effect data.

### 2.3 Study selection

The literature search, screening, and data extraction were independently performed by two reviewers (LB and LY), followed by cross-checking for consistency. Discrepancies were resolved through group discussions and consensus. Reference management was conducted using EndNote X9 software. After removing duplicate records, titles, and abstracts were screened to eliminate irrelevant studies. Full texts of the remaining articles were then assessed according to the inclusion and exclusion criteria to determine the final set of eligible studies.

### 2.4 Data extraction

Data from eligible studies were independently extracted by two authors (LB and LY) using a categorized form. Discrepancies were resolved through consensus within the review team. The extracted data included: first author, year of publication, country/location of study, sample size, age, gender, study design, depression/anxiety assessment tools, outcome indicators, method of DII assessment, and pooled effect size of the included studies.

### 2.5 Risk of bias assessment

Risk of bias was independently assessed by two reviewers, followed by cross-checking. Discrepancies were addressed through group discussion and consensus. The Newcastle-Ottawa Scale (NOS) was used to assess the quality of cohort studies, with a maximum score of 9 stars. Studies were rated as high quality (≥7 stars), moderate quality (4–6 stars), or low quality (≤3 stars) based on their scores (17). For cross-sectional studies, risk of bias was assessed using the Agency for Healthcare Research and Quality (AHRQ) checklist. Studies were categorized as high quality (8–11 points), moderate quality (4–7 points), or low quality (0–3 points) based on their total score (18).

### 2.6 Statistical methods

Meta-analysis was conducted using Stata 18.0. Hazard ratios (HRs) and odds ratios (ORs) were treated as approximately equivalent to relative risks (RRs), following standard practice in meta-analyses of relatively rare outcomes such as depression or anxiety (19, 20). This approximation is supported by previous methodological studies, which have demonstrated that for rare events, ORs and HRs closely approximate RRs and can thus be combined in pooled analyses (21, 22). All included studies were using ORs and their corresponding 95% CIs as the effect measures. We acknowledge that this approach may introduce some degree of heterogeneity, which was accounted for by applying a random-effects model when appropriate.

Statistical heterogeneity across studies was assessed using the *Q* test and *I*^2^ statistic. If *p* ≥ 0.05 and *I*^2^ ≤ 50%, heterogeneity was deemed small and acceptable, and a fixed-effects model was applied; otherwise, a random-effects model was used. Subgroup analyses were performed based on study design, gender, age, region, and survey methods to further explore sources of heterogeneity. Sensitivity analysis was conducted to evaluate the robustness of the pooled estimates, including an additional analysis pooling studies that reported DII as a continuous variable. Publication bias was examined using Egger’s test and visually assessed through funnel plots.

A nonlinear dose–response relationship between DII and the risk of depression or anxiety was assessed using the generalized least squares estimation method, along with a restricted cubic spline model with three knots placed at the 10th, 50th, and 90th percentiles of the exposure distribution (23, 24). A Wald test was used to assess the presence of nonlinear. If *p* < 0.05, a nonlinear dose–response relationship was considered present; otherwise, a linear relationship was assumed. Model fit was also assessed to ensure the validity of the results.

## 3 Results

### 3.1 Literature screening

**Figure 1:**
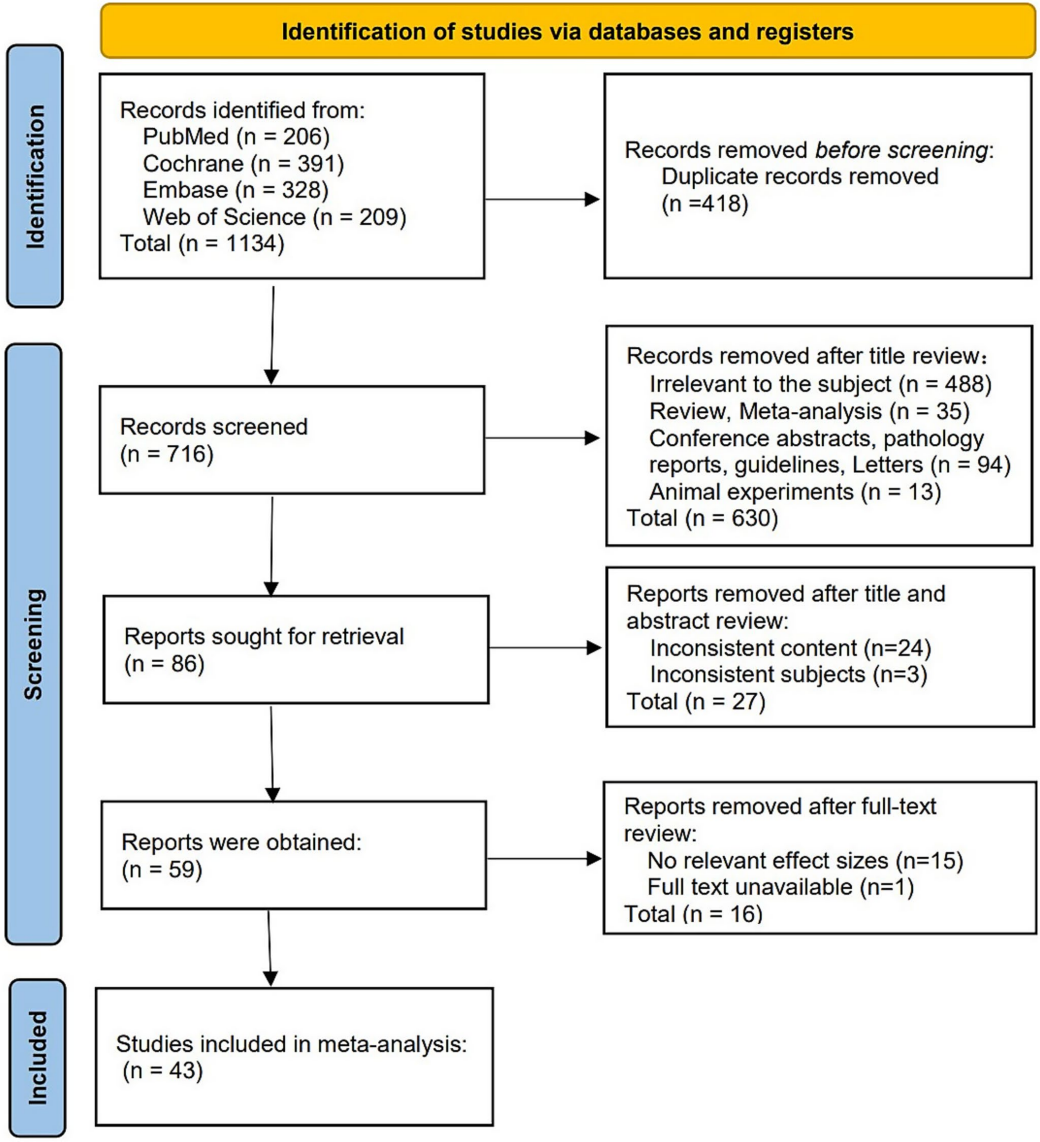
Literature screening flow chart. The PRISMA-based diagram illustrates the process of study identification, screening, eligibility, and inclusion, from initial search results to final studies included in the meta-analysis. Reproduced from Page et al. (16), licensed under CC BY 4.0.

A total of 1,134 articles were initially retrieved. After removing duplicates and excluding irrelevant studies, 43 studies were ultimately included in the analysis. The flowchart of the literature screening process and results is shown in Figure 1.

### 3.2 Characteristics of included studies

All included studies were observational in design, comprising 11 cohort studies and 32 cross-sectional studies, published between 2015 and 2025. The studies were conducted across four continents: Asia (China, Iran, Turkey, South Korea, and the United Arab Emirates; 12 studies), North America (United States; 20 studies), Europe (United Kingdom, France, Spain, and Ireland; 10 studies), and Australia (1 study). In all 43 studies, DII scores were analyzed as categorical variables.

### 3.3 Risk of bias assessment of included studies

The risk of bias assessment indicated that the overall quality of the included studies was moderate to high, with 21 studies rated as high quality and 22 studies as moderate quality. Among the 43 studies, depression was the primary outcome in 33 studies, anxiety in one study, and both depression and anxiety in nine studies. In these studies, participants were categorized into groups based on their DII scores: the group with the highest DII score represented the most pro-inflammatory diet, while the group with the lowest DII score represented the most anti-inflammatory diet. The characteristics and quality assessment of the included studies are summarized in Table 1.

**Table 1:** Characteristics of the included studies and results of bias risk assessment.

Author	Year	Country /Location	Study design	Age (y)	Sample size	Outcome measurement tool	DII assessment method	Outcome indicator	Risk of bias score
Bergmans (53)	2017	USA	Cross-sectional	>20	11,592	PHQ-9	24HR	Depression, anxiety	7*
Wirth (54)	2017	USA	Cross-sectional	Depression: 45.7 No depression: 47.0	18,875	PHQ-9	24HR	Depression	8*
Phillips (55)	2018	Ireland	Cross-sectional	50–69	1,992	CES-D	FFQ	Depression, anxiety	8*
Shivappa (56)	2018	Iran	Cross-sectional	15–18	300	DASS-21	FFQ	Depression	9*
Açik (57)	2019	Türkiye	Cross-sectional	19–24	134	ZSDS	24HR	Depression	9*
Salari-Moghaddam (58)	2019	Iran	Cross-sectional	36.3 ± 7.8	3,363	HADS	FFQ	Depression, anxiety	8*
Ghazizadeh (10)	2020	Iran	Cross-sectional	35–65	7,083	BAI、BDI-II	FFQ	Depression, anxiety	7*
Molud (59)	2020	Iran	Cross-sectional	25–65	4,630	Professional Physician Screening	FFQ	Depression	8*
Shin (60)	2020	South Korea	Cross-sectional	≥19	15,929	PHQ-9	NI	Depression	7*
Ma (61)	2021	China	Cross-sectional	66.3 ± 0.3	1,865	GDS	FFQ	Depression	7*
Salari-Moghaddam (62)	2021	Iran	Cross-sectional	36.3	3,363	HADS	FFQ	Depression, anxiety	8*
Shakya (63)	2021	Austria	Cross-sectional	56.6 ± 13.6	1,743	CES-D	FFQ	Depression	6*
Attlee (64)	2022	UAE	Cross-sectional	20.3 ± 1.8	260	DASS-21	24HR	Depression, anxiety	9*
Chen (65)	2022	China	Cross-sectional	20–80	220	CES-D	24HR	Depression	7*
Azarmanesh (66)	2022	USA	Cross-sectional	Before menopause: 35.9 ± 9.6 After menopause: 62.9 ± 9.9	4,908	PHQ-9	24HR	Depression	6*
Jiang (67)	2022	USA	Cross-sectional	60.3 ± 14.8	2,770	PHQ-9	24HR	Depression	7*
Li (68)	2022	China	Cross-sectional	64	2,022	GDS	FFQ	Depression	7*
Sun (69)	2022	USA	Cross-sectional	≧60	2,550	PHQ-9	interview	Depression	7*
Xiao (70)	2022	USA	Cross-sectional	70 ± 7.0	10,956	PHQ-9	24HR	Depression	7*
Zhang (71)	2022	USA	Cross-sectional	≧18	27,447	PHQ-9	24HR	Depression	7*
Ding (72)	2023	USA	Cross-sectional	20–80	12,788	PHQ-9	24HR	Depression	6*
Luo (73)	2023	USA	Cross-sectional	≧18	10,951	PHQ-9	24HR	Depression	6*
Wang (74)	2023	USA	Cross-sectional	≧20	21,785	PHQ-9	24HR	Depression	7*
Navab (75)	2024	Iran	Cross-sectional	20–50	262	DASS-21	FFQ	Depression, anxiety	8*
Wang (76)	2024	USA	Cross-sectional	47.2 ± 0.3	21,865	PHQ-9	24HR	Depression	7*
Sánchez-Villegas (77)	2015	Spain	Cohort study	38.3	15,093	Professional Physician Screening	FFQ	Depression	7#
Akbaraly (78)	2016	UK	Cohort study	61.0 ± 5.9	4,246	CES-D	FFQ	Depression	7#
Adjibade (30)	2017	France	Cohort study	35–60	3,523	CES-D	24HR	Depression	7#
Shivappa (79)	2018	USA	Cohort study	61.4 ± 9.2	3,608	CES-D	24HR	Depression	7#
Adjibade (80)	2019	France	Cohort study	≧18	26,730	CES-D	24HR	Depression	6#
Bizzozero-Peroni (81)	2022	Spain	Cohort study	Cohort 1: 71.5 ± 5.5 Cohort 2: 71.4 ± 4.2	Cohort 1: 1,627 Cohort 2: 1,579	Self-Report, Diagnostic Record, GDS	Dietary history	Depression	7#
Zheng (82)	2024	UK	Cohort study	40–70	2,785	Self-Report, Diagnostic Record	24HR	Anxiety	8#
Ma (83)	2024	USA	Cross-sectional	≧20	19,612	PHQ-9	24HR	Depression	8*
Zhai (84)	2024	USA	Cohort study	56.1 ± 8.0	152,853	Self-report, Diagnosis record	24HR	Depression	7#
Zhang (85)	2024	USA	Cross-sectional	63–85	1,239	PHQ-9	24HR	Depression	7*
Duan (86)	2025	USA	Cross-sectional	33–64	32,210	PHQ-9	NI	Depression	7*
Fu (87)	2025	UK	Cohort study	59.0 ± 8.1	164,863	Case records	24HR	Depression, anxiety	8#
Huang (88)	2025	USA	Cross-sectional	57.8	4,232	PHQ-9	24HR	Depression	8*
Pang (89)	2025	UK	Cohort study	56.5 ± 8.1	189,835	Case records, PHQ-9, GAD-7	24HR	Depression, anxiety	7#
Ren (90)	2025	USA	Cross-sectional	49.7 ± 16.4	14,305	PHQ-9	24HR	Depression	6*
You (91)	2025	USA	Cross-sectional	≧20	7,553	PHQ-9	24HR	Depression	7*
Zheng (92)	2025	USA	Cross-sectional	≧20	20,446	PHQ-9	24HR	Depression	6*
Zhou (93)	2025	UK	Cohort study	37–73	55,799	Case records	24HR	Depression	8#

*, Risk of bias assessment for cross-sectional studies; #, Risk of bias assessment for cohort studies; 24HR, 24-Hour Dietary Recall; BAI, Beck Anxiety Inventory; BDI-II, Beck Depression Inventory-II; CES-D, Center for Epidemiological Studies Depression Scale; DASS-21, Depression Anxiety Stress Scales - 21 Items; FFQ, Food Frequency Questionnaire; GDS, Geriatric Depression Scale; HADS, Hospital Anxiety and Depression Scale; NI, no information; PHQ-9, Patient Health Questionnaire; UAE, United Arab Emirates; ZSDS, Zung Self-Rating Depression Scale.

### 3.4 Meta-analysis

#### 3.4.1 Association between DII and the risk of depression, with exploratory findings on anxiety

**Figure 2:**
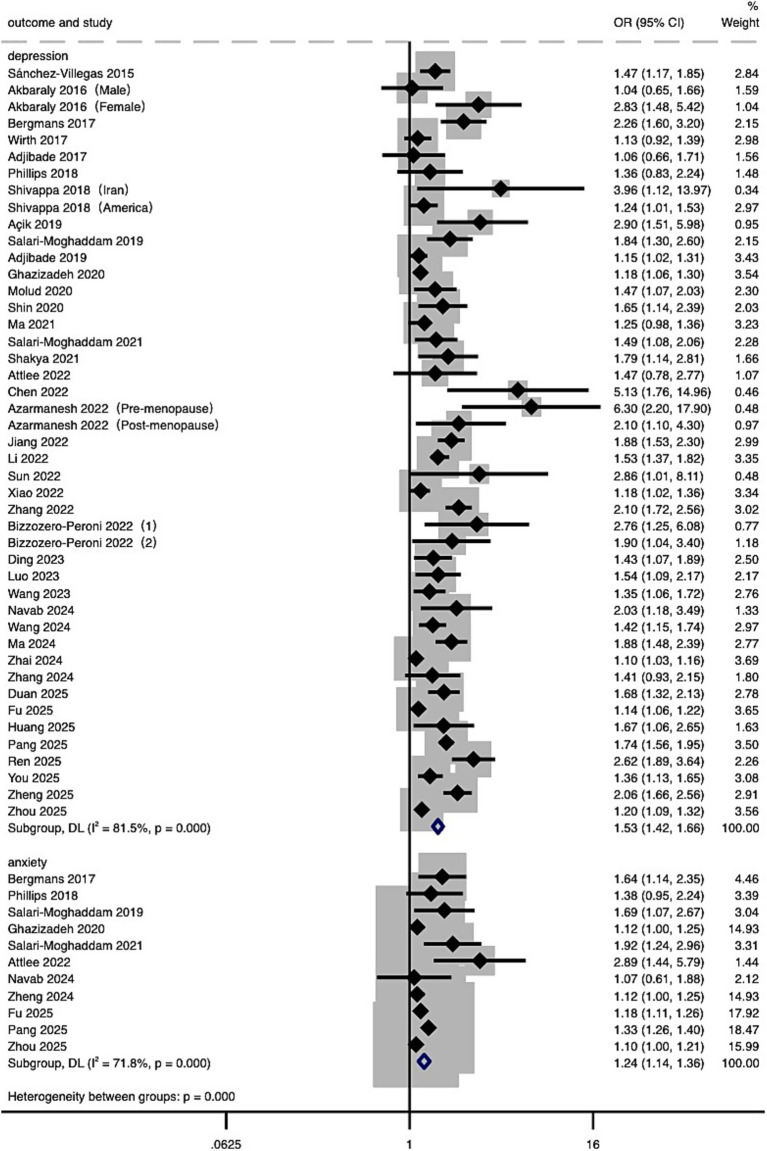
Forest plot of the relationship between DII and risk of depression and anxiety. Effect estimates (ORs and 95% CIs) are pooled from included studies using a random-effects model. Separate estimates for depression and anxiety are presented.

A total of 43 studies were included in the meta-analysis examining the association between DII and the risk of depression. The analysis compared individuals with the highest DII scores (most pro-inflammatory diet) to those with the lowest scores (most anti-inflammatory diet). Due to substantial heterogeneity (*I*^2^ = 81.5%, *p* < 0.001), a random-effects model was employed. The pooled analysis showed that individuals with the most pro-inflammatory diets had a 53% higher risk of depression (OR = 1.53, 95% CI: 1.42 to 1.66). As a sensitivity analysis, when pooling studies that reported DII as a continuous variable, a significant positive association was also observed (OR = 1.10, 95% CI: 1.06 to 1.15), though with higher heterogeneity (*I*^2^ = 91.6%, *p* < 0.001) (Supplementary Figure S1). Exploratory analyses of a smaller subset of studies suggested a 24% increased risk of anxiety (OR = 1.24, 95% CI: 1.14 to 1.36) (Figure 2).

#### 3.4.2 Subgroup analysis

To explore the potential sources of heterogeneity, subgroup analyses were conducted based on study design, gender, age, region, dietary assessment methods, depression assessment tools, and body mass index (BMI). As shown in Table 2, the association between higher DII and increased depression risk remained consistent across all subgroups, although the magnitude of the effect varied slightly. Although the magnitude of association varied slightly. While subgroup–specific estimates were informative, no statistically significant differences between subgroups were formally tested. Overall, these results suggest that the association between DII and depression risk is robust across various populations and methodological strata.

**Table 2:** Results of subgroup analysis of the relationship between DII and depression.

Basis for grouping	Articles included (*N*)	Effect model	OR (95% CI)	Heterogeneity test	
				*p*	*I*^2^ (%)
Study design					
Cohort	10	Random	1.31 (1.16–1.47)	<0.001	84.1
Cross-sectional	32	Random	1.63 (1.49–1.79)	<0.001	72.1
Gender					
Male	16	Random	1.24 (1.15–1.33)	0.001	57.3
Female	22	Random	1.28 (1.20–1.37)	<0.001	65.4
Age (y)					
18 ≦ age < 60	14	Random	1.42 (1.28–1.57)	<0.001	72.1
≥ 60	11	Random	1.32 (1.19–1.46)	0.002	62.6
Location					
Asia	12	Random	1.55 (1.34–1.79)	0.001	64.6
Europe	10	Random	1.32 (1.17–1.49)	<0.001	84.1
North America	19	Random	1.64 (1.45–1.86)	<0.001	75.3
Dietary assessment methods					
FFQ	13	Random	1.43 (1.28–1.60)	0.009	53.6
24HR	25	Random	1.54 (1.39–1.71)	<0.001	86.9
BMI (kg/m^2^)					
<25	6	Fixed	1.15 (1.11–1.19)	0.604	<0.001
≥25	6	Random	1.24 (1.14–1.34)	0.001	73.3
Depression assessment tools					
PHQ-9	19	Random	1.67 (1.48–1.89)	<0.001	73.8
CES-D	6	Random	1.44 (1.14–1.82)	0.007	65.9
DASS-21	3	Fixed	1.91 (1.29–2.83)	0.370	<0.001

24HR, 24-Hour Dietary Recall; BMI, Body Mass Index; CES-D, Center for Epidemiological Studies Depression Scale; DASS-21, Depression Anxiety Stress Scales - 21 Items; FFQ, Food Frequency Questionnaire; PHQ-9, Patient Health Questionnaire.

#### 3.4.3 Dose–response meta-analysis of DII and the risk of depression

**Figure 3:**
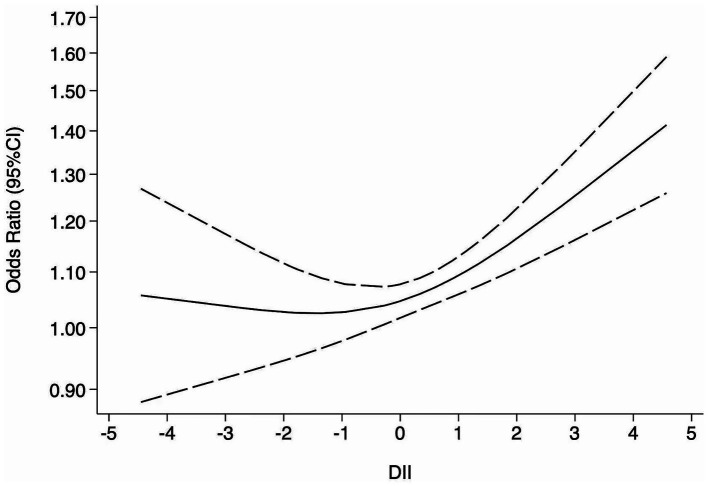
Dose–response meta-analysis of DII and risk of depression. The solid line represents the estimated odds ratio (OR), and the dashed lines indicate the 95% confidence intervals (CI) derived from the restricted cubic spline model.

The restricted cubic spline model (Figure 3) demonstrated a statistically significant non-linear association between DII and depression risk (p for non-linearity = 0.0019). The curve remained relatively flat for DII values below approximately 0, indicating no significant increase in risk, but rose steadily once DII exceeded this threshold, reaching an OR of about 1.20–1.35 (95% CI: 1.03 to 1.58) at DII levels of 3–4. This non-linear pattern was consistent with the study-specific estimates at different DII levels shown in the forest plot (Supplementary Figure S2), in which most effect estimates for DII values below 0 were close to unity, whereas significantly elevated risks were observed at higher DII levels. For anxiety, only two studies provided data suitable for dose–response analysis; therefore, no further analysis was conducted.

#### 3.4.4 Publication bias and sensitivity analysis

**Figure 4:**
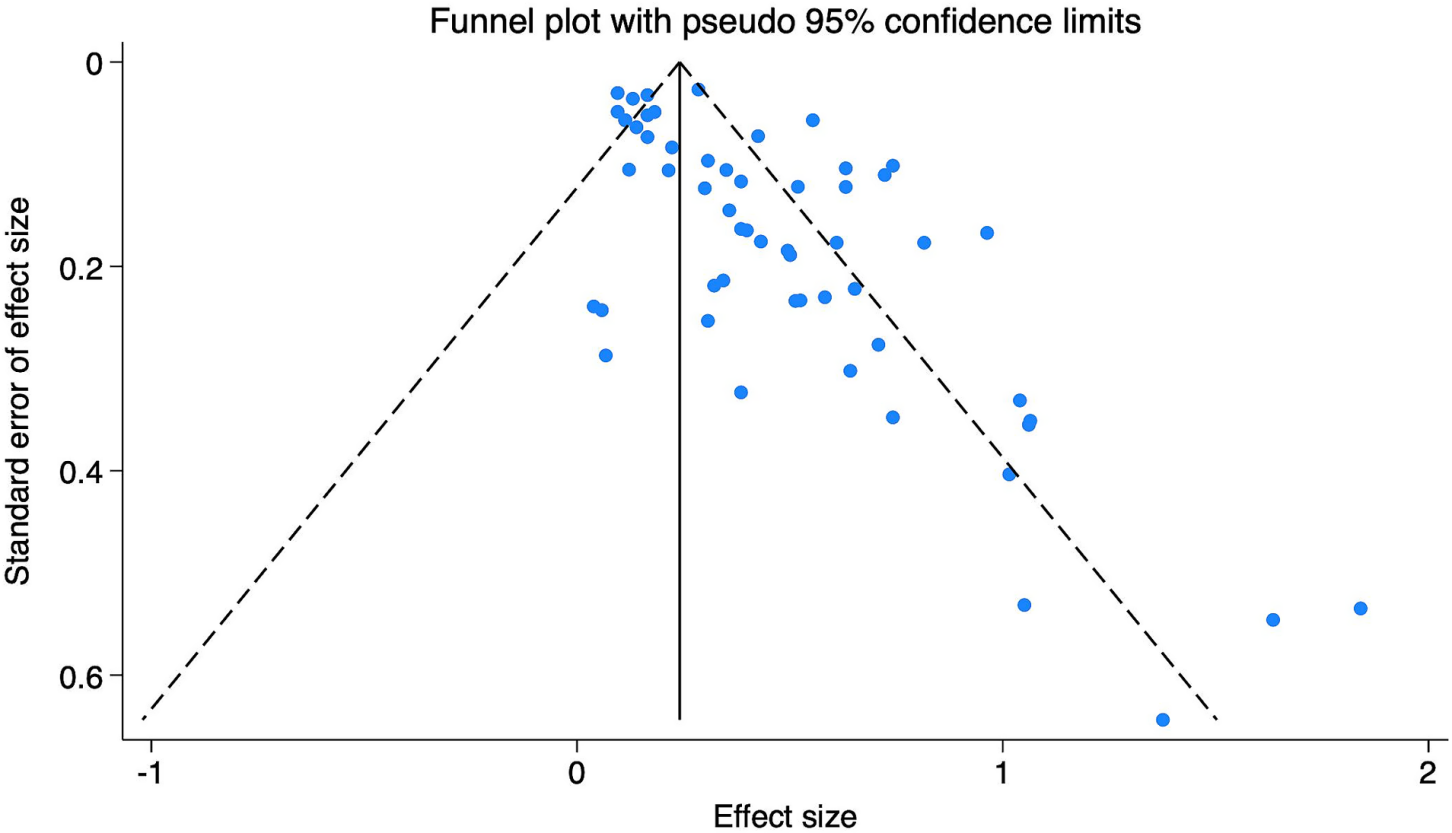
Funnel plot of research on the relationship between DII and depression. The funnel plot illustrates the distribution of individual study estimates relative to their standard errors. Asymmetry may suggest potential publication bias.

Publication bias and sensitivity analysis were conducted using Stata 18.0 software. The funnel plot (Figure 4) appeared asymmetrical, suggesting the presence of potential publication bias, which was further supported by Egger’s test (*p* < 0.05). Subgroup funnel plots are provided in the Supplementary Figures S3–S8 for visual appraisal only. To assess the robustness of the findings, a sensitivity analysis was performed by systematically removing one study at a time. No significant changes were observed in the overall effect estimates, indicating that the results were stable and reliable.

## 4 Discussion

### 4.1 Relationship between DII and the risk of depression

#### 4.1.1 Main findings from the meta-analysis

This meta-analysis revealed a significant association between higher DII scores and an increased risk of depression and, to a lesser extent, anxiety (see Figure 2). Specifically, the dose–response analysis indicated that when DII scores exceed 0, depression risk increases progressively (see Figure 3). Importantly, the sensitivity analysis using studies that assessed DII as a continuous variable also confirmed a significant positive association, further supporting the robustness of our findings despite methodological heterogeneity (Supplementary Figure S1). Collectively, evidence from the categorical main analysis, dose–response modeling, and the continuous-exposure sensitivity analysis suggests that the association is not an artifact of a particular operationalization of DII or a single analytic contrast. Heterogeneity was expected given differences in instruments and populations, but it did not overturn the overall direction of the association (see Section 4.1.2).

A brief mechanistic bridge provides context for these observational findings. Pro-inflammatory diets are linked to up-regulated systemic inflammatory mediators (e.g., CRP, IL-6, TNF-*α*) that can influence neurotransmitter metabolism and neuropeptide signaling; diet also modulates the microbiota–gut–brain axis, and inflammatory activation may heighten HPA-axis reactivity and oxidative stress (see Section 4.2). These interlocking pathways provide biologically credible routes through which higher dietary inflammatory load may relate to depressive symptoms.

These results suggest that dietary modifications - such as increasing the intake of anti-inflammatory foods (e.g., fish, whole grains, legumes, fresh fruits, and vegetables) and reducing consumption of pro-inflammatory items (e.g., processed meats, red meats, refined carbohydrates, and saturated fats) – may have potential mental health benefits (25).

However, these results should be interpreted with caution. As most included studies were observational, causal relationships cannot be definitively established (26). Furthermore, the possibility of bidirectionality—where mood states may also shape dietary choices—cannot be excluded, and clarifying directionality will require longitudinal and interventional designs (see Section 4.2).

#### 4.1.2 Subgroup analysis

Subgroup analyses demonstrated a consistent positive association between DII and depression across all examined strata, including study design, gender, age, region, dietary assessment methods, depression assessment tools, and BMI (see Table 2). Although no formal statistical tests were conducted to compare subgroups, the direction and strength of the association remained generally stable, reinforcing the robustness of the findings.

The association appeared stronger in cross-sectional studies compared to cohort studies, possibly reflecting differences in study design and the potential influence of residual confounding or reverse causation in cross-sectional analysis (27).

Gender-stratified analyses showed a slightly stronger association in females than in males, in line with prior findings suggesting that hormonal fluctuations or menopausal transitions may increase susceptibility to depression in females (28, 29). However, this interpretation remains tentative. Interestingly, Adjibade et al. (30) reported a significant association between higher DII and depressive symptoms in males in a large cohort study of 3,523 participants aged 35–60. This apparent discrepancy may be partially explained by confounders such as smoking, which is more prevalent in males and has been associated with increased depression risk (31).

Age-stratified analysis indicated a stronger association in adults under 60 years compared to older adults. This may reflect the earlier onset and higher prevalence of depression in younger and middle-aged populations, although the mechanisms behind this age-related pattern are not fully understood (32).

Additionally, the method of dietary assessment also influenced effect estimates. Studies employing 24-h dietary recall (24HR) demonstrated higher estimates than those using Food Frequency Questionnaires (FFQs). This variation may be due to differences in recall accuracy, food group resolution, or the timeframe captured by each method. While 24HR provides detailed intake data over a short period, FFQs may better represent habitual dietary patterns but may be subject to greater recall bias (33).

The depression assessment tool also contributed to some variation in effect estimates. Studies employing the Patient Health Questionnaire-9 (PHQ-9) tended to show higher associations, the Center for Epidemiologic Studies Depression scale (CES-D) yielded moderate estimates, and the 21-item Depression Anxiety Stress Scales (DASS-21) produced the strongest associations. These differences may reflect variability in instrument sensitivity, score thresholds, or the weighting of somatic versus cognitive symptoms (34–36). Nevertheless, all tools consistently demonstrated a positive association between DII and depression.

Overall, these subgroup findings underscore the robustness of the main results and suggest that the relationship between dietary inflammation and depression may be modified by demographic, methodological, and lifestyle factors. Future research should aim to further investigate these potential moderators, using prospective study designs and standardized assessment tools to clarify causality and strengthen the evidence base.

### 4.2 Mechanisms underlying the relationship between DII and depression

Currently, the biological mechanisms linking DII to depression remain incompletely elucidated. However, accumulating evidence suggests that mental disorders are associated with elevated levels of inflammatory biomarkers, such as interleukin-6 (IL-6), tumor necrosis factor-alpha (TNF-*α*), and C-reactive protein (CRP) (37, 38). These pro-inflammatory cytokines may regulate neurotransmitter metabolism and neuropeptide concentrations, thereby contributing to the development and progression of depressive symptoms (39). Increasing evidence highlights the critical role of diet in modulating the gut microbiota, which, in turn, influences the gut-brain axis - a key pathway implicated in the development and maintenance of neuropsychiatric disorders (40). Dietary patterns can profoundly affect microbial composition and metabolic output, thereby influencing immune responses, neuroinflammation, and brain function (41). This “diet–microbiota–gut–brain axis” provides a compelling biological basis for the link between dietary inflammation and mental health outcomes (42).

Additionally, excessive secretion of inflammatory factors may hyperactivate the hypothalamic–pituitary–adrenal (HPA) axis, alter monoamine neurotransmitter levels, and damage neuronal cells through oxidative stress (43). Diet quality plays a key role in modulating immune function and systemic inflammation levels, which may, in turn, trigger or exacerbate depressive symptoms (44). Collectively, these pathways offer biologically plausible mechanisms linking higher DII (greater dietary inflammatory load) to depressive phenotypes, while acknowledging that causal direction cannot yet be established.

Consistent with this possibility, several studies indicate that psychological distress can shift dietary preferences toward more processed, pro-inflammatory patterns (45, 46). Papier et al. found that stress could prompt individuals, particularly students, to choose more processed foods (46). Similarly, D. J. Korczak et al. observed that children with major depressive disorder consumed fewer healthy foods (47). These findings highlight the need for longitudinal studies to disentangle the directionality of the association and determine whether dietary interventions can causally improve mental health outcomes.

### 4.3 Publication bias and its implications

Egger’s test and the funnel plot suggested potential publication bias in studies examining the association between DII and depression. This may reflect a tendency for studies with statistically significant or positive results to be more likely to be published, while those with null or negative findings remain unpublished. Such bias can lead to an overestimation of the true effect size in meta-analyses and may limit the generalizability of findings (48).

Although the pooled association between DII and depression remained statistically significant, the presence of publication bias underscores the need for cautious interpretation. Assessment of publication bias within subgroups is inherently underpowered and difficult to interpret; therefore, the subgroup funnel plots are presented as descriptive evidence to enhance transparency, and inferences are based primarily on the overall analysis. It underscores the importance of including unpublished data, gray literature, or pre-registration of protocols in future research to mitigate bias and improve the robustness of evidence (48). In addition, prospective cohort studies with rigorous methodology may help validate these findings and provide a more accurate estimate of the diet–mental health relationship.

## 5 Strengths and limitations of the study

### 5.1 Strengths of the study

To our knowledge, this meta-analysis offers a comprehensive and up-to-date assessment of the association between dietary inflammatory potential and depression. It incorporates a broad range of recent studies and applies dose–response analyses to clarify the nature of the relationship. Subgroup analyses by demographic and methodological factors (e.g., age, gender, region, dietary and depression assessment methods, and BMI) offer a nuanced understanding of potential sources of heterogeneity and strengthen the robustness of the findings. Exploratory analyses were also performed on anxiety outcomes in a smaller subset of studies.

### 5.2 Limitations of the study

While this meta-analysis provides valuable insights, several limitations should be acknowledged. First, all included studies were observational in design, which limits causal inferences and leaves open the possibility of residual confounding despite statistical adjustments (26). Second, potential publication bias was identified, which may affect the reliability and generalizability of the findings (49). Third, dietary intake was predominantly assessed through self-reported tools such as FFQ and 24HR, which are prone to recall bias and measurement error, thereby affecting the accuracy of DII estimation (50). Fourth, most studies were conducted in specific geographic and cultural contexts, which may restrict broader applicability (51). Fifth, the assumption that ORs, HRs, and RRs are approximately equivalent for rare outcomes may introduce minor estimation bias (52). Finally, to ensure methodological consistency, we primarily included studies reporting DII as categorical variables, which may have reduced comprehensiveness compared to integrating continuous data (13).

Further research should prioritize well-designed prospective studies with standardized dietary assessment and consistent DII calculation methods across diverse populations to strengthen the evidence base and validate these findings.

## 6 Conclusion

This meta-analysis indicates that higher DII scores are associated with an increased risk of depression, with evidence of a nonlinear relationship. Exploratory findings suggested a similar trend for anxiety, but the limited number of studies warrants cautious interpretation. These results support the potential mental health benefits of anti-inflammatory dietary patterns. Given the observational nature of the included studies and the presence of moderate heterogeneity and possible publication bias, further well-designed prospective studies are needed to confirm these associations and clarify underlying mechanisms. These findings may help inform dietary guidelines and public health strategies to promote mental well-being through nutritional interventions.

## Data Availability

The original contributions presented in the study are included in the article/Supplementary material, further inquiries can be directed to the corresponding authors.
