# Supplementary material for: Nonlinear dose–response relationship between dietary inflammatory index and risk of depression: a systematic review and meta-analysis
Source: Front Nutr. 2025 Sep 12;12:1645789. doi: 10.3389/fnut.2025.1645789 (PMC12465630; doi:10.3389/fnut.2025.1645789)
Supplement: Supplementary file 1 [file Table_1.DOCX]

**SEARCH STRATEGY**

**2025.8.9**

**PUBMED**

Search: ((("Depression" [Mesh]) OR "Anxiety" [Mesh]) OR ((((((depression[Title/Abstract]) OR (anxiety[Title/Abstract])) OR (Depressive Symptom[Title/Abstract])) OR (Emotional

Depression[Title/Abstract])) OR (Angst[Title/Abstract])) OR (Nervousness[Title/Abstract]))) AND ((((((dietary inflammatory index[Title/Abstract]) ) OR (DI[Title/Abstract])) OR (inflammatory diet[Title/Abstract])) OR (anti-inflammatory diet[Title/Abstract])) OR (dietary score[Title/Abstract])) **206**

**Web of Science**

(depression (Topic) or anxiety (Topic) or "Depressive Symptom" (Topic) or "Emotional Depression" (Topic) or Angst (Topic) or Nervousness (Topic)) AND ("dietary inflammatory index" (Topic) or DIl (Topic) or "Inflammatory diet" (Topic) or "anti-inflammatory diet" (Topic) or "dietary score" (Topic) ) **209**

**Embase**

(("anxiety'/exp OR 'anxiety') OR ("depression"/exp OR 'depression') OR ('depressive symptom':ti, ab,kw OR 'emotional depression':ti, ab,kw OR angst:ti, ab,kw OR nervousness:ti, ab,kw)) AND ("dietary inflammatory index':ti,ab,kw OR dit:ti, ab,kw OR 'inflammatory diet':ti,ab,kw OR 'anti-inflammatory diet':ti,ab,kw OR 'dietary score':ti,ab,kw)  **328**

**Cochrane:**

("dietary inflammatory index"'):ti.ab.kw OR (DIl):ti,ab.kw OR ("inflammatory diet"'):ti,ab.kw OR ("anti-inflammatory diet"):ti,ab. kw OR ("dietary score"):ti, ab.kw **391**
