# Supplementary material for: Nonlinear dose–response relationship between dietary inflammatory index and risk of depression: a systematic review and meta-analysis
Source: Front Nutr. 2025 Sep 12;12:1645789. doi: 10.3389/fnut.2025.1645789 (PMC12465630; doi:10.3389/fnut.2025.1645789)
Supplement: Supplementary file 2 [file Table_2.DOCX]

Supplementary Material

# Supplementary Figures

For more information on Supplementary Material and for details on the different file types accepted, please see [here](https://www.frontiersin.org/guidelines/author-guidelines#supplementary-material).

**
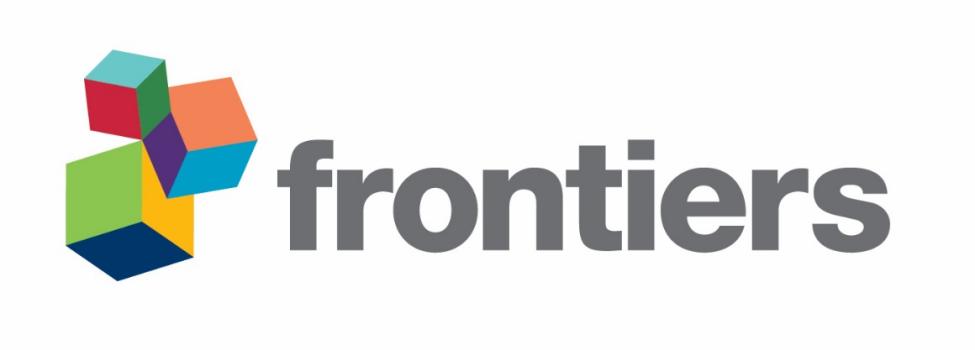
**

**Supplementary Figure S1.**


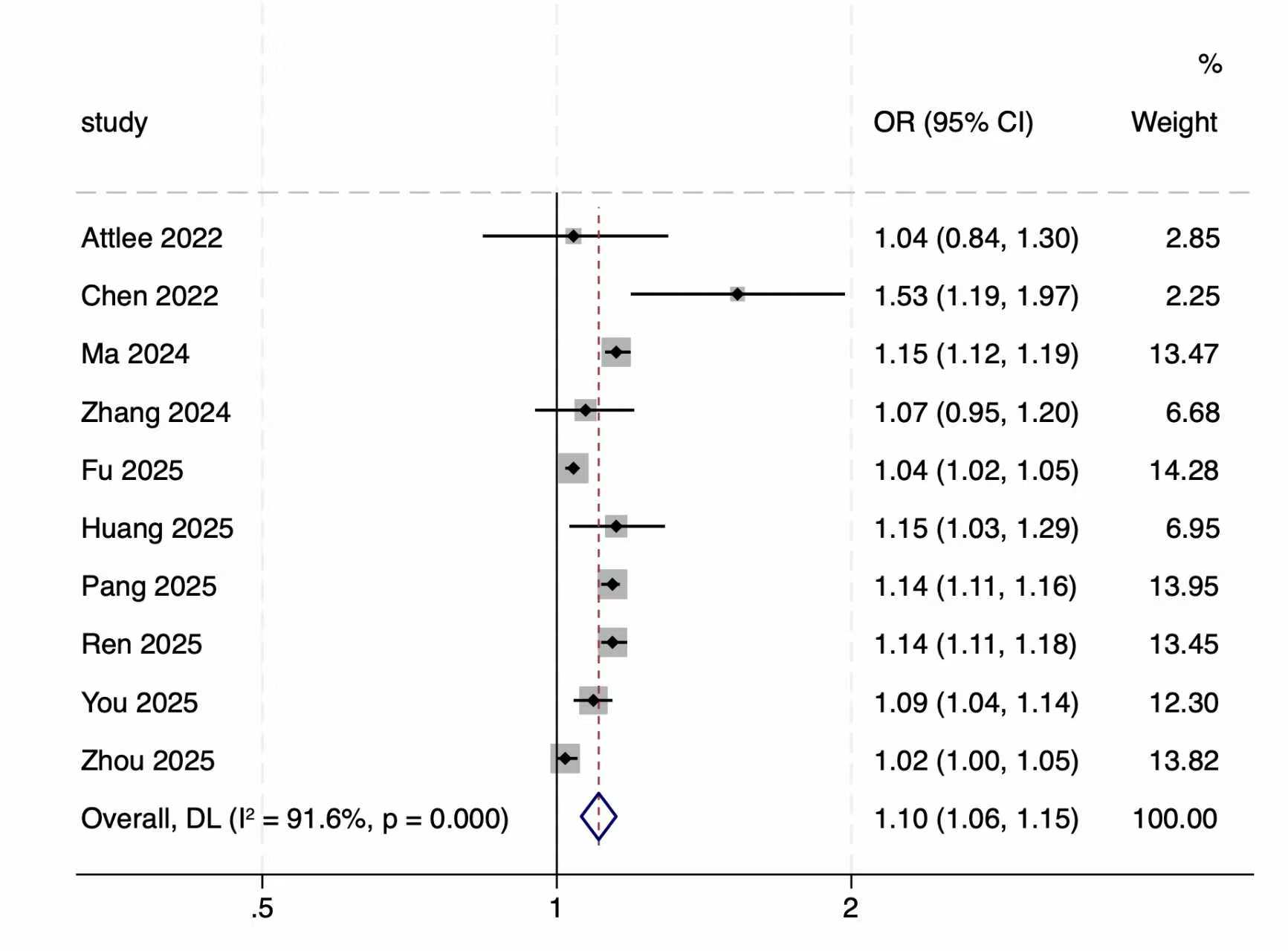


**Supplementary Figure S1.**

**Forest plot of the association between continuous Dietary Inflammatory Index (DII) and risk of depression.**

The figure presents pooled odds ratios (ORs) and 95% confidence intervals (CIs) for the association between Dietary Inflammatory Index (DII, modeled as a continuous variable) and risk of depression. The summary estimate was calculated using a random-effects model. Squares represent study-specific effect estimates, with the size proportional to study weight; horizontal lines denote 95% CIs; and the diamond indicates the overall pooled effect size.

**Supplementary Figure S2.**

**Supplementary Figure S2.**

**Forest plot of dose–response relationship between DII and risk of depression.**

Each square represents the odds ratio (OR) for a specific DII level in an individual study, with the size proportional to the study weight. Horizontal lines indicate 95% confidence intervals (CIs). DII values (dose) are shown in parentheses next to study names; negative values indicate more anti-inflammatory diets, while positive values indicate more pro-inflammatory diets.

**Supplementary Figure S3.**

| **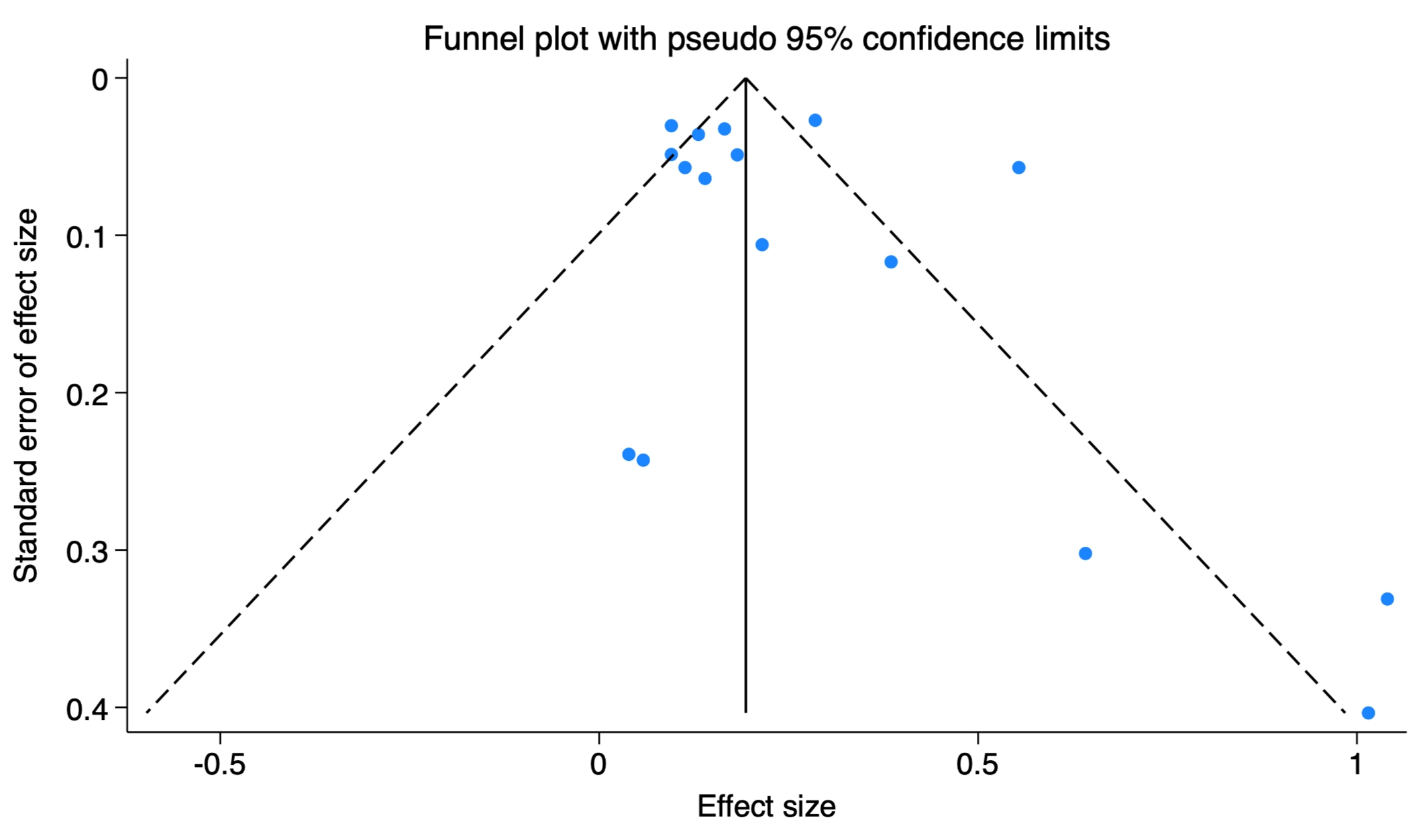** |
| --- |
| **(A) Cohort studies (n =10)** |
| **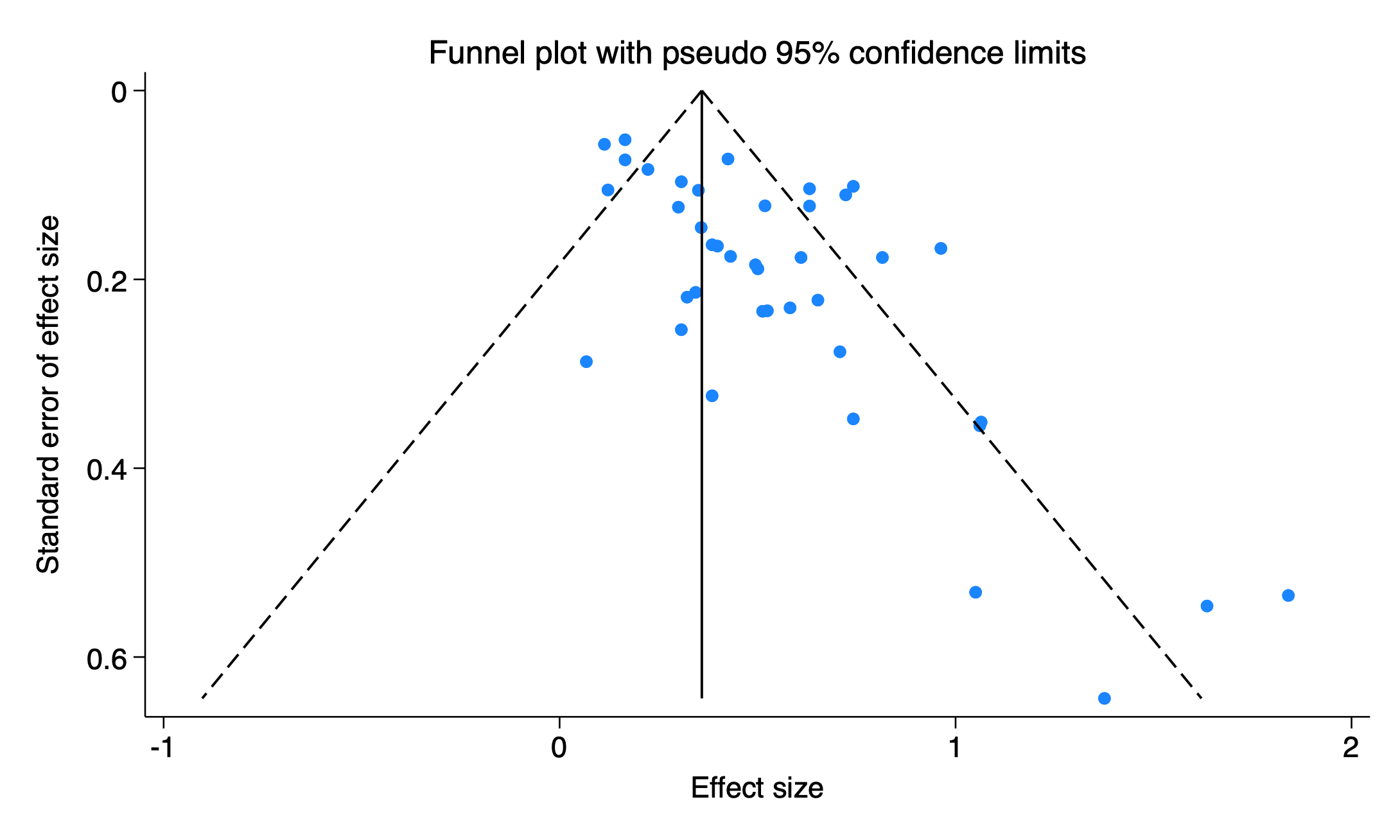** |
| **(B) Cross-sectional studies (n = 32)** |

**Supplementary Figure S3. Funnel plots for publication bias by study design.**

Each dot represents one study. The x-axis shows the effect size (OR) and the y-axis the standard error. The solid vertical line denotes the pooled effect estimate; the dashed diagonal lines indicate pseudo 95% confidence limits.

**Supplementary Figure S4.**

| **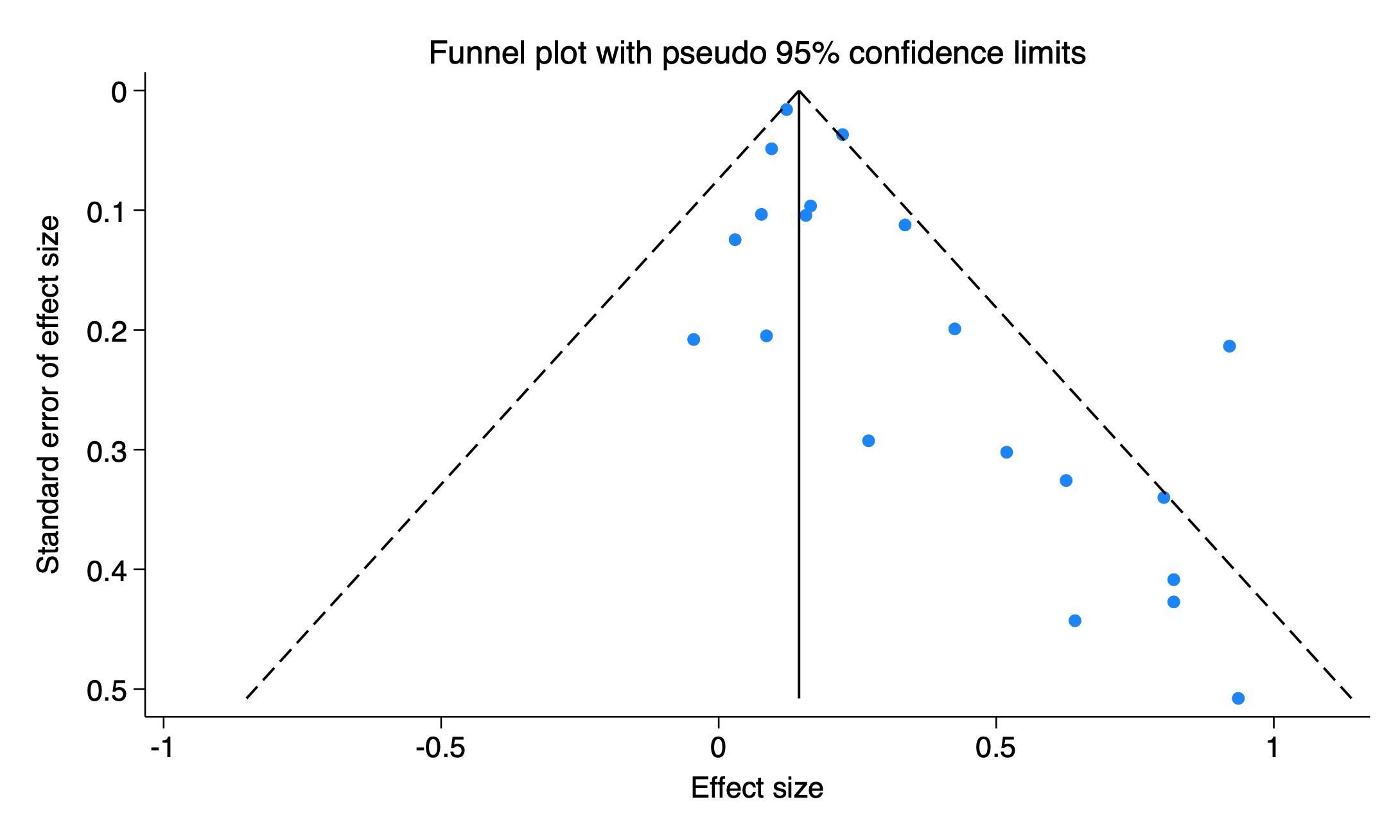** |
| --- |
| **(A) Male (n =16)** |
| **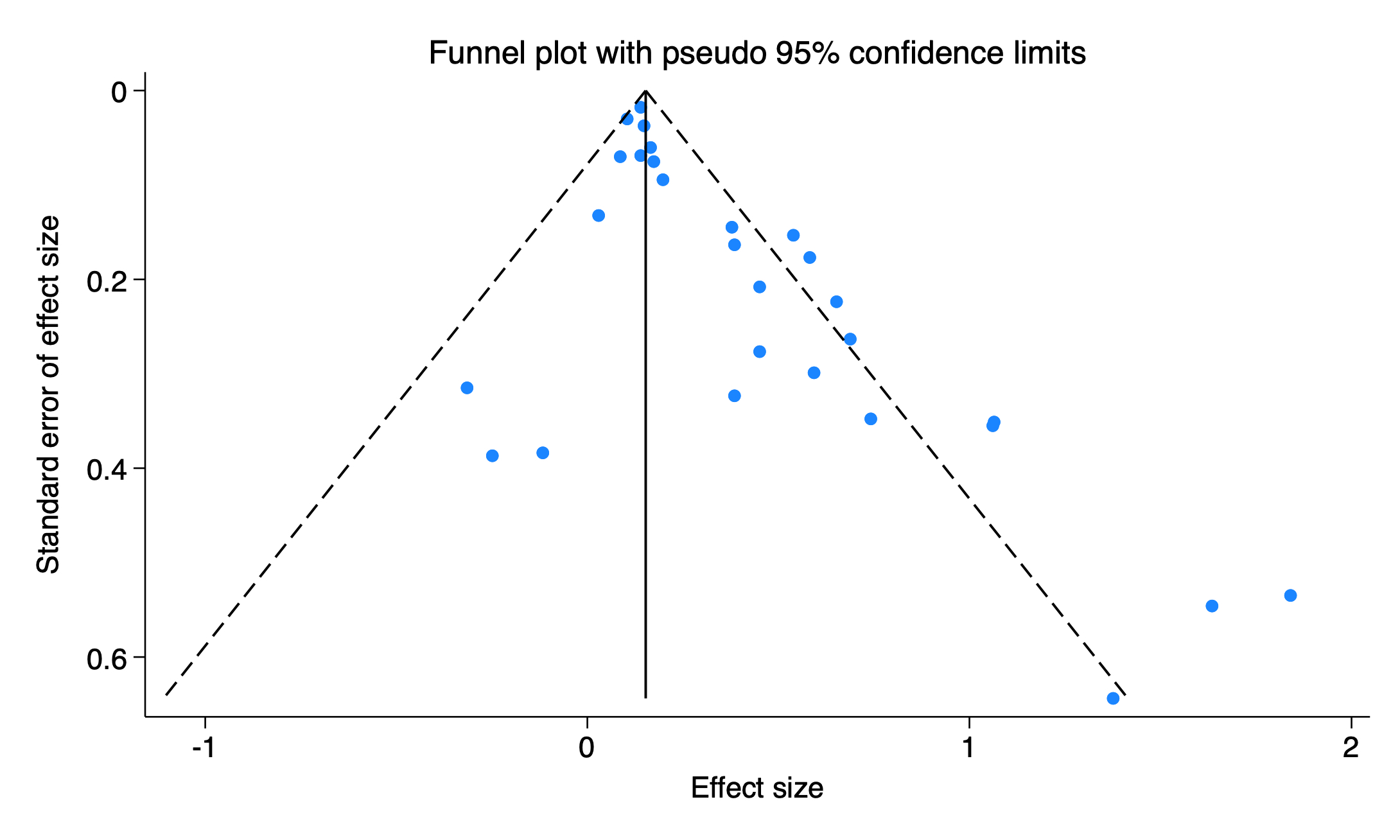** |
| **(B) Female (n = 22)** |

**Supplementary Figure S4. Funnel plots for publication bias by gender.**

Each dot represents one study. The x-axis shows the effect size (OR) and the y-axis the standard error. The solid vertical line denotes the pooled effect estimate; the dashed diagonal lines indicate pseudo 95% confidence limits.

**Supplementary Figure S5.**

| **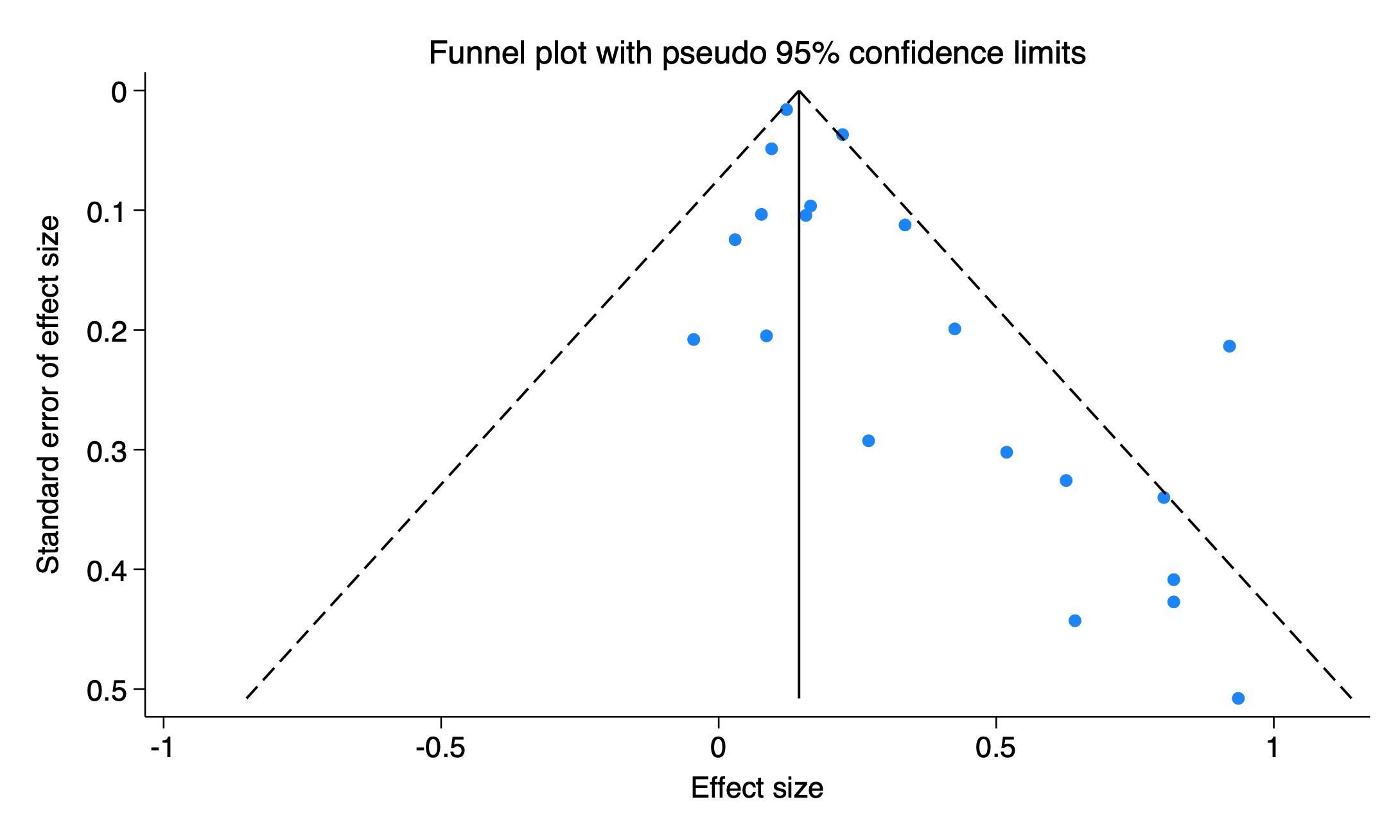** |
| --- |
| **(A) 18 ≦ age < 60 (n =14)** |
| **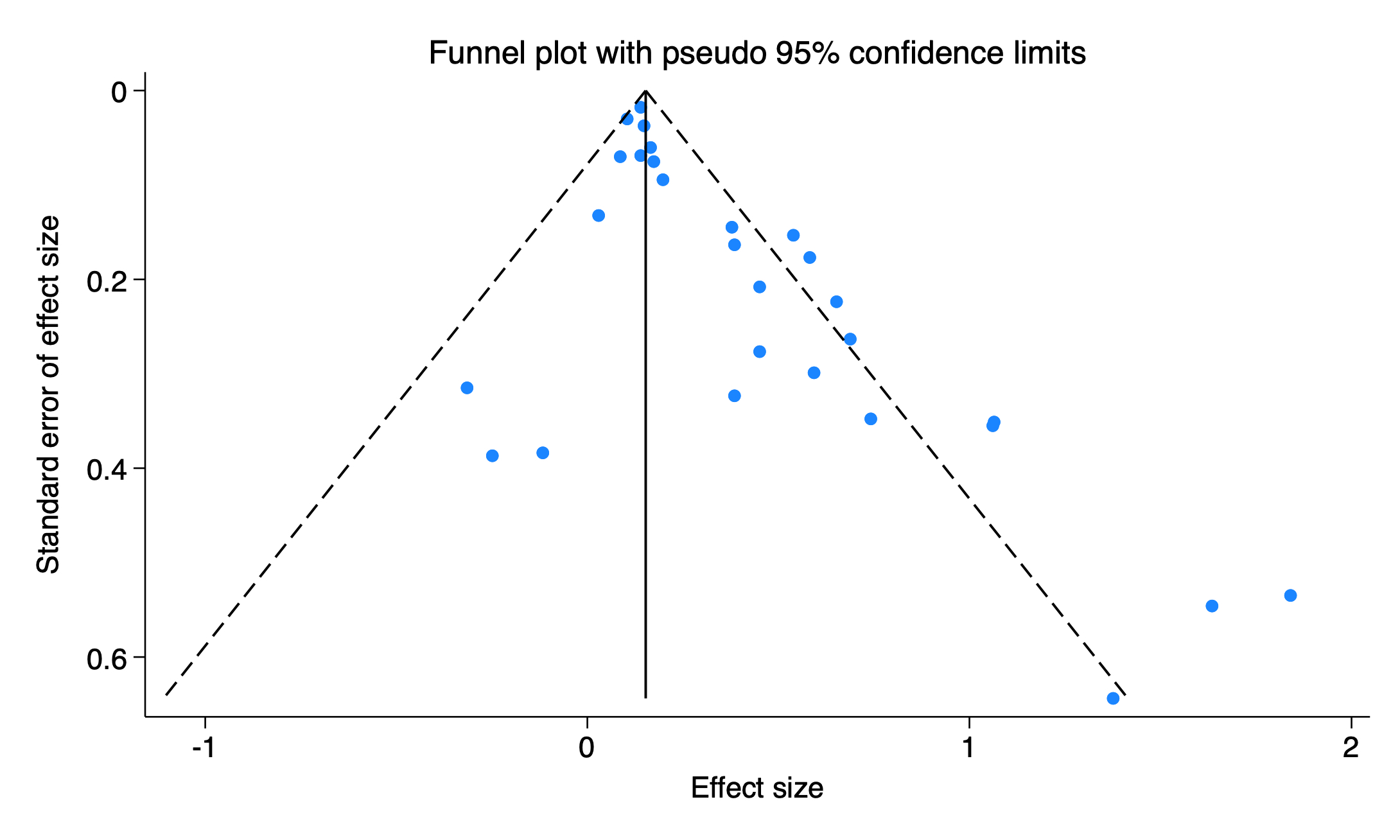** |
| **(B) age≥ 60 (n = 11)** |

**Supplementary Figure S5. Funnel plots for publication bias by age.**

Each dot represents one study. The x-axis shows the effect size (OR) and the y-axis the standard error. The solid vertical line denotes the pooled effect estimate; the dashed diagonal lines indicate pseudo 95% confidence limits.

**Supplementary Figure S6.**

| **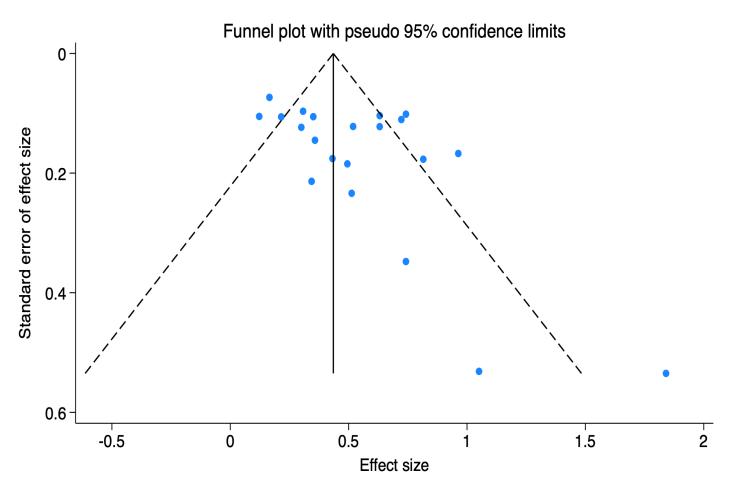** | **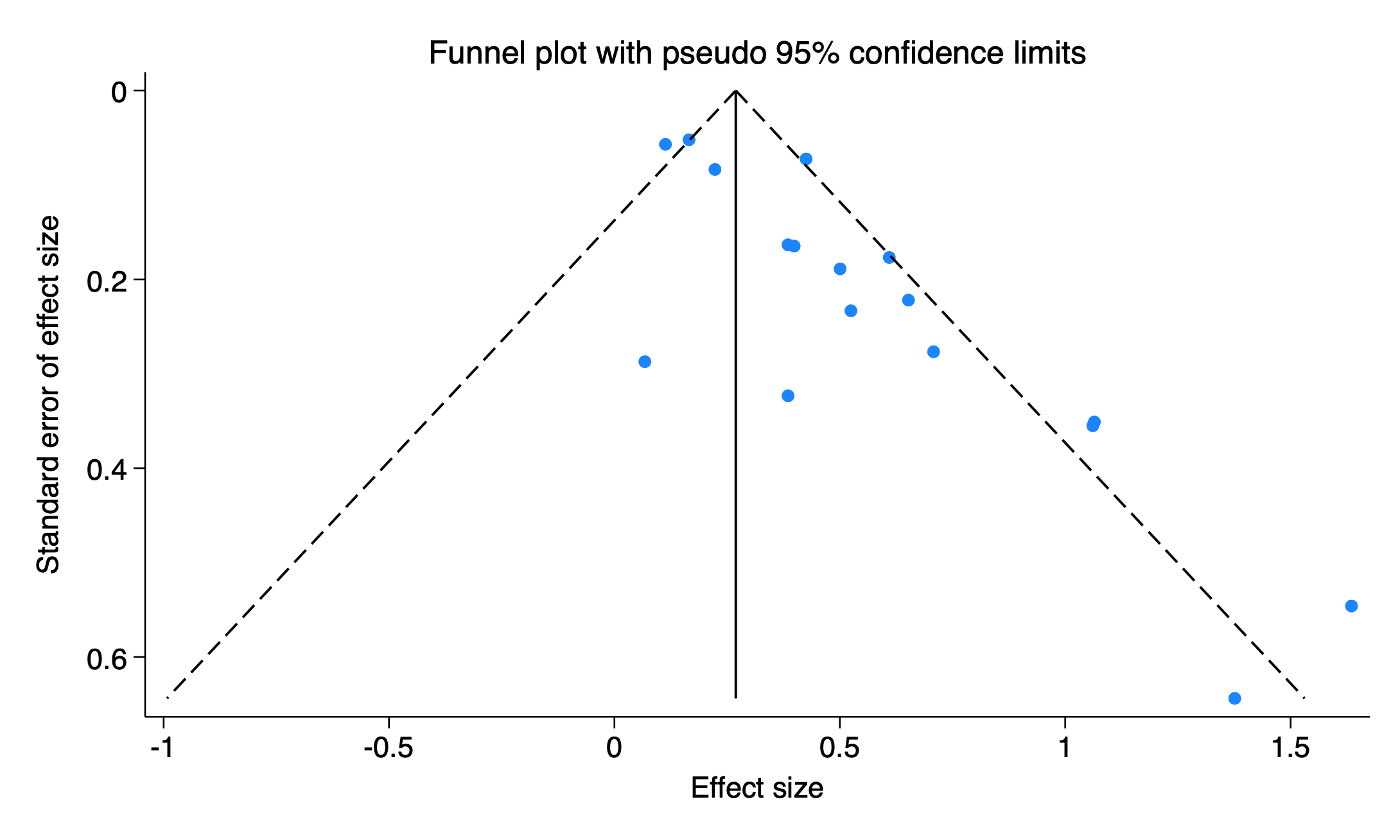** |
| --- | --- |
| **(A) North America (n =19)** | **(B) Asia (n = 12)** |
| **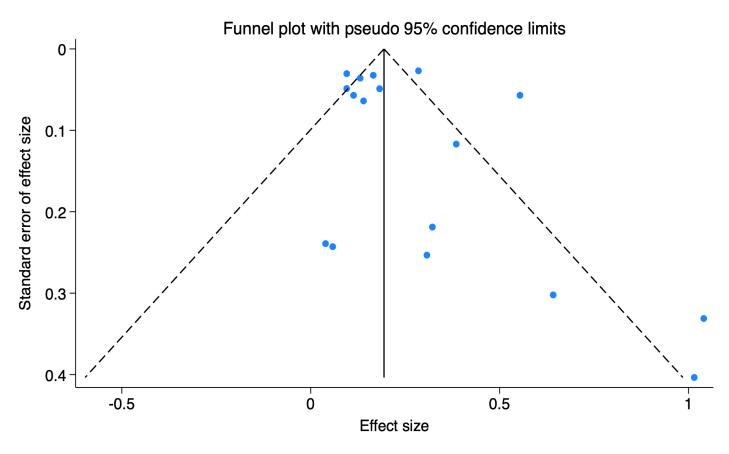** |  |
| **(C) Europe (n =10)** |  |

**Supplementary Figure S6. Funnel plots for publication bias by location.**

Each dot represents one study. The x-axis shows the effect size (OR) and the y-axis the standard error. The solid vertical line denotes the pooled effect estimate; the dashed diagonal lines indicate pseudo 95% confidence limits.

**Supplementary Figure 7.**

| **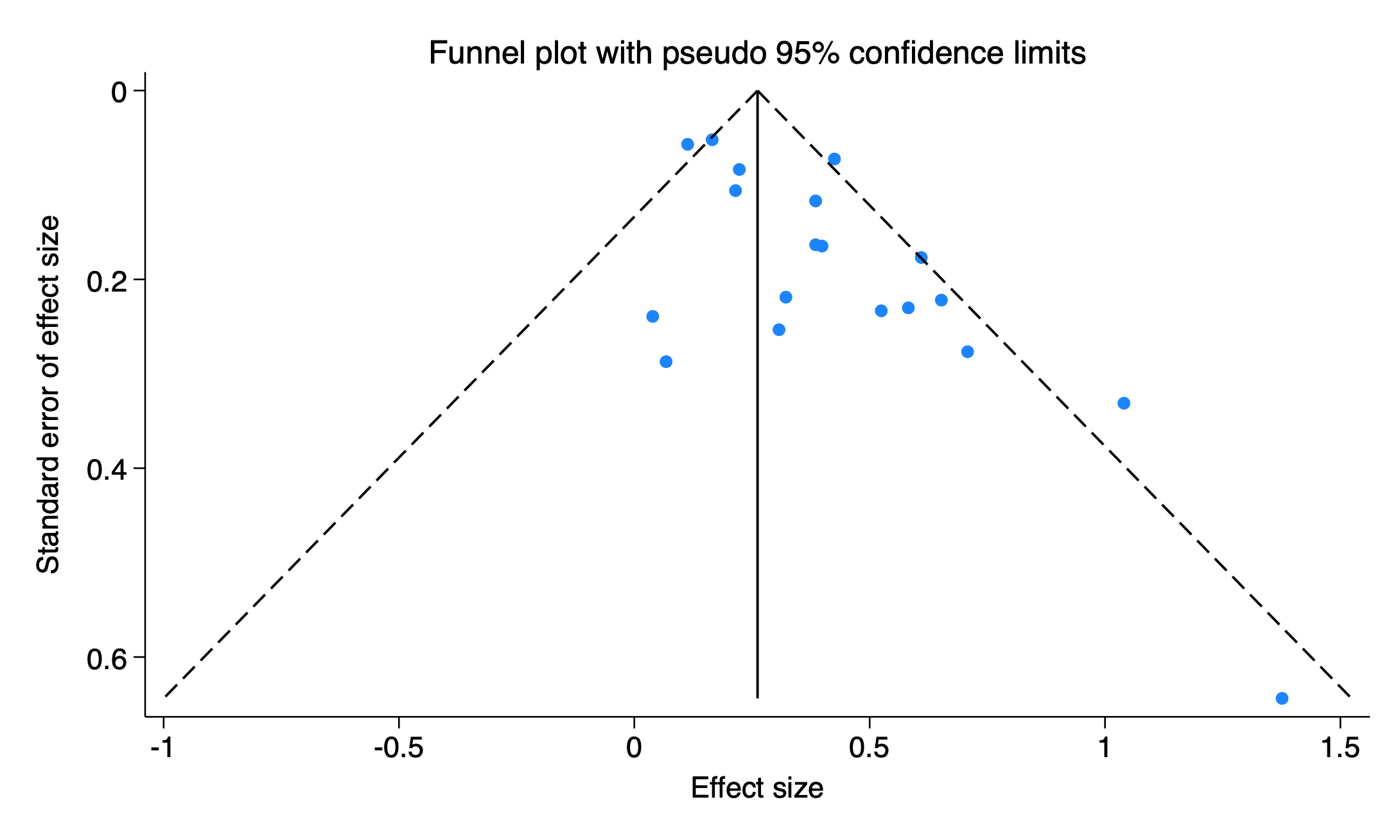** |
| --- |
| **(A) FFQ (n =13)** |
| **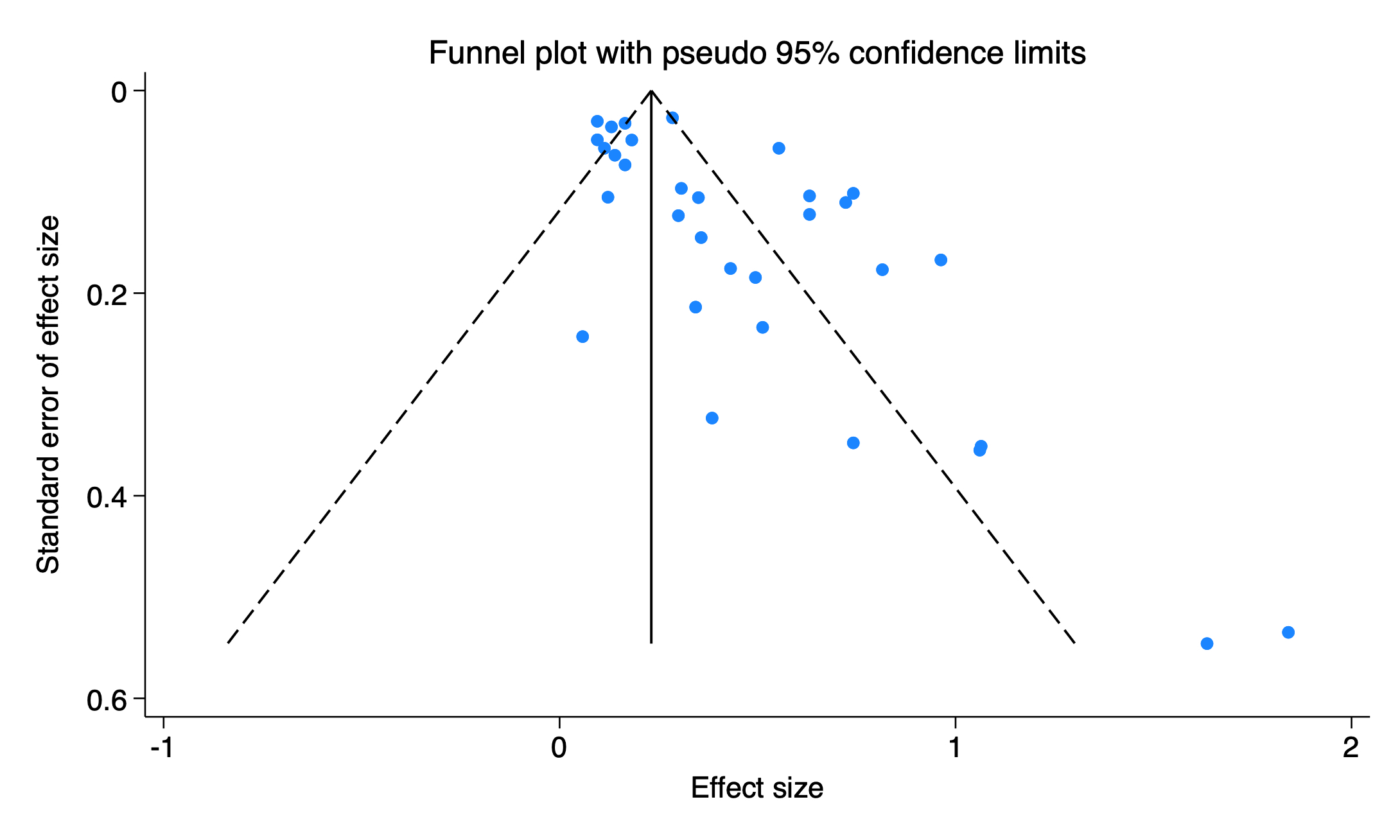** |
| **(B) 24HR (n = 25)** |

**Supplementary Figure S7. Funnel plots for publication bias by dietary assessment methods.**

Each dot represents one study. The x-axis shows the effect size (OR) and the y-axis the standard error. The solid vertical line denotes the pooled effect estimate; the dashed diagonal lines indicate pseudo 95% confidence limits.

**Supplementary Figure 8.**

| **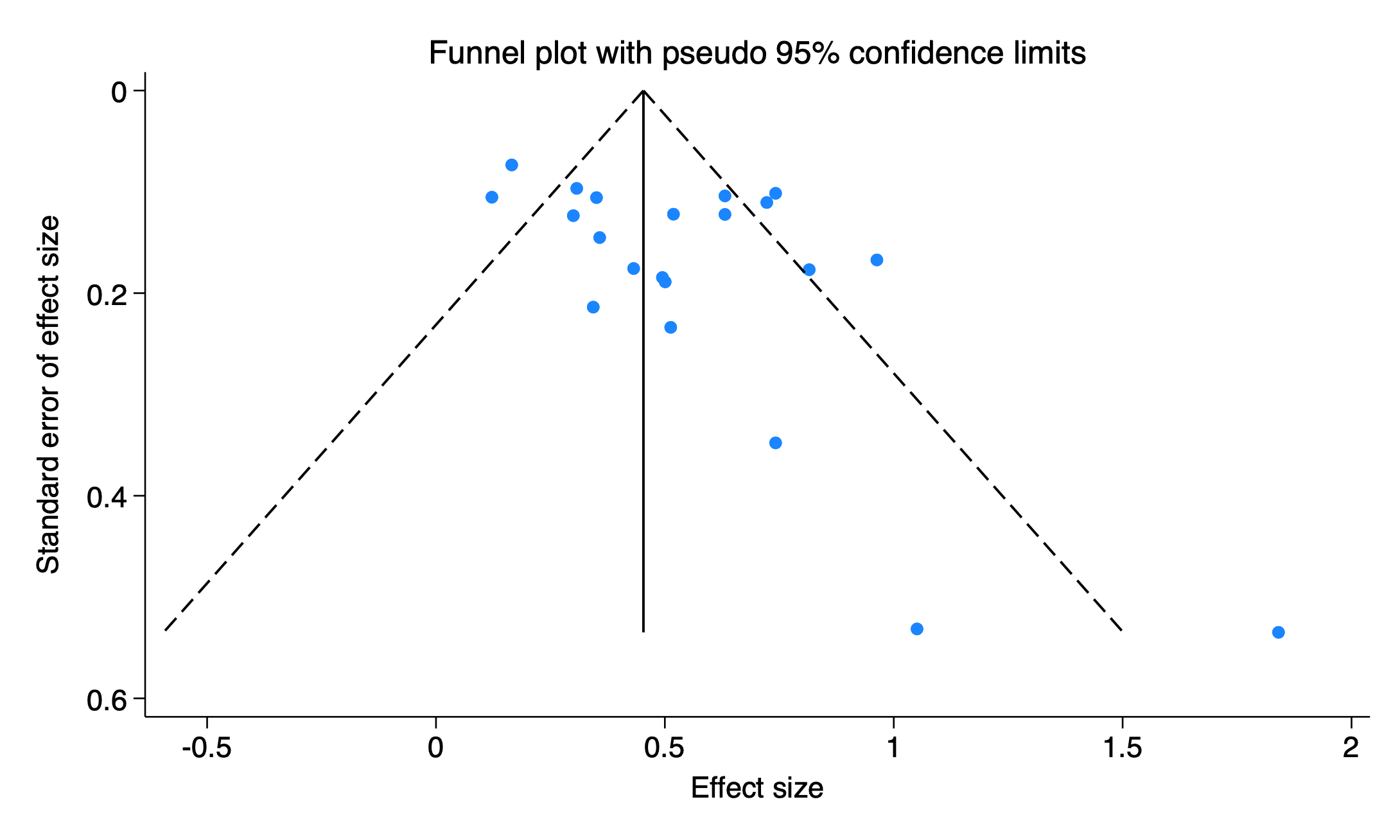** |
| --- |
| **PHQ-9 (n =19)** |

**Supplementary Figure S8. Funnel plots for publication bias by depression assessment tools-PHQ-9 .**

Each dot represents one study. The x-axis shows the effect size (OR) and the y-axis the standard error. The solid vertical line denotes the pooled effect estimate; the dashed diagonal lines indicate pseudo 95% confidence limits.
